# Respiratory Epithelial Cells Respond to *Lactobacillus plantarum* but Provide No Cross-Protection against Virus-Induced Inflammation

**DOI:** 10.3390/v13010002

**Published:** 2020-12-22

**Authors:** Eric Mai, Caroline M. Percopo, Ajinkya R. Limkar, Albert C. Sek, Michelle Ma, Helene F. Rosenberg

**Affiliations:** 1 Inflammation Immunobiology Section, Laboratory of Allergic Diseases, National Institute of Allergy and Infectious Diseases, National Institutes of Health, Bethesda, MD 20892, USA; eric.mai96@gmail.com (E.M.); percopoc@niaid.nih.gov (C.M.P.); limka001@umn.edu (A.R.L.); albertcsek@gmail.com (A.C.S.); michelle.ma@nih.gov (M.M.); 2 Weill Cornell Medical College, New York, NY 10021, USA; 3 Laboratory of Malaria and Vector Research, National Institute of Allergy and Infectious Diseases, Twinbrook III, National Institutes of Health, Rockville, MD 20852, USA; 4 Medical Scientist Training Program, University of Wisconsin School of Medicine and Public Health, Madison, WI 53726, USA; 5 Merck Research Laboratories, South San Francisco, CA 94080, USA; 6 Genetic Immunotherapy Section, Laboratory of Clinical Microbiology and Immunology, National Institute of Allergy and Infectious Diseases, National Institutes of Health, Bethesda, MD 20892, USA

**Keywords:** *Lactobacillus plantarum*, Influenza A, inflammation, cytokine, pattern recognition receptor

## Abstract

Virus-induced inflammation plays a critical role in determining the clinical outcome of an acute respiratory virus infection. We have shown previously that the administration of immunobiotic *Lactobacillus plantarum* (Lp) directly to the respiratory tract prevents lethal inflammatory responses to subsequent infection with a mouse respiratory virus pathogen. While Lp-mediated protective responses involve non-redundant contributions of both Toll-like receptor 2 (TLR2) and NOD2, the cellular basis of these findings remains unclear. Here, we address the impact of Lp and its capacity to suppress inflammation in virus-infected respiratory epithelial cells in two cell culture models. We found that both MLE-12 cells and polarized mouse tracheal epithelial cells (mTECs) were susceptible to infection with Influenza A and released proinflammatory cytokines, including CCL2, CCL5, CXCL1, and CXCL10, in response to replicating virus. MLE-12 cells express NOD2 (81 ± 6.3%) and TLR2 (19 ± 4%), respond to Lp, and are TLR2-specific, but not NOD2-specific, biochemical agonists. By contrast, we found that mTECs express NOD2 (81 ± 17%) but minimal TLR2 (0.93 ± 0.58%); nonetheless, mTECs respond to Lp and the TLR2 agonist, Pam2CSK4, but not NOD2 agonists or the bifunctional TLR2-NOD2 agonist, CL-429. Although MLE-12 cells and mTECS were both activated by Lp, little to no cytokine suppression was observed in response to Lp followed by virus infection via a protocol that replicated experimental conditions that were effective in vivo. Further study and a more complex approach may be required to reveal critical factors that suppress virus-induced inflammatory responses.

## 1. Introduction

Influenza (Inf) and Respiratory Syncytial Virus (RSV) together with Severe Acute Respiratory Syndrome Coronavirus 2 (SARS-CoV-2) are currently the most important respiratory virus pathogens currently circulating worldwide. Among the numerous problems associated with ongoing detection and control measures, it has become clear that some individuals infected with these viruses are asymptomatic or experience mild symptoms only, while others develop severe and potentially life-threatening disease [1,2,3,4,5,6].

Virus-induced inflammation plays a critical role in determining the clinical outcome of acute respiratory virus infection [7,8,9,10,11,12]. We and others have explored this issue extensively with the mouse pneumovirus pathogen, pneumonia virus of mice (PVM; [13,14,15]). PVM is a rodent-specific virus of the same virus family (Pneumoviridae) and genus (Pneumovirus) as RSV; our acute infection model with PVM phenocopies the severe outcomes that have been described in RSV-infected human infants and children [1,16]. Among the findings that suggest a crucial role for the inflammatory response in the pathogenesis of PVM disease, Walsh and colleagues [17] reported that administration of a sphingosine-1-phosphate (S1P) receptor 1 (S1P1R) agonist blunted the cytokine storm and provided protection against the lethal sequelae of PVM infection. Likewise, Bonville and colleagues [18,19] documented a similar role for the chemokine CCL3, and Bondue and colleagues [20] identified the chemerin receptor ChemR23 as a critical factor underlying inflammatory pathogenesis.

We have explored the role of inflammation in PVM infection in a series of studies that feature administration of the immunobiotic Gram-positive bacterial species *Lactobacillus plantarum* (Lp) directly to the respiratory tracts of wild-type and gene-deleted mice [21,22,23,24,25,26]. We found that administration of Lp directly to the respiratory tract provides full protection against the lethal sequelae of PVM infection that lasts for up to six months [21]. Despite minimal impact on virus replication and clearance, administration of Lp resulted in profound suppression of virus-induced inflammation in the lungs and the airways with reduced levels of proinflammatory chemokines, including CCL2, CXCL1, and CXCL10 [21,22,23,24,25,26]. Administration of various *Lactobacillus* spp. to the respiratory tracts also limits inflammatory pathology in mouse models of Influenza A (Inf A) and RSV [27,28,29,30,31]. Taken together, these findings may explain, in part, the results of Rosas-Salazar and colleagues [32], who reported a relative overabundance of *Lactobacillus* spp. in the upper airways of previously healthy infants who did not go on to develop wheezing after recovery from acute RSV infection.

We have also found that Lp priming of the respiratory tract elicits local production of proinflammatory cytokines via TLR2/NOD2 dependent pathways [24,33] and that mice devoid of both pattern recognition receptors are not fully protected by Lp against the lethal sequelae of PVM infection [24]. The cellular and molecular basis of these critical responses has not yet been clarified [22,25,26]. Interestingly, and despite their characterized role in the process of trained innate immunity [34], monocytes’ recruitment to the respiratory tract is not required for Lp-mediated protection against lethal PVM infection [26].

Here, we studied the responses of isolated respiratory epithelial cells to Lp and the potential of this immunobiotic agent to suppress virus-induced inflammation in this setting.

## 2. Materials and Methods

### 2.1. Mice

Wild-type (WT) C57BL/6 and BALB/c mice (6–12 weeks old) were purchased from Charles River Laboratories (Frederick, MD). Toll-like receptor 2 gene-deleted mice (*Tlr2^−/−^*) on a BALB/c background were maintained on-site at the National Institute of Allergy and Infectious Diseases/National Institutes of Health (NIAID/NIH) 14BS vivarium. All mouse studies were approved by the National Institute of Allergy and Infectious Diseases Animal Care Committee and performed in accordance with Animal Study Protocol LAD-8E.

### 2.2. MLE-12 Cells

The mouse lung epithelial cell line MLE-12 [35] was purchased from the American Type Culture Collection (ATCC; Manassas, VA, USA, CRL-2110). Low-passage cells were cultured in 50/50 Dulbecco’s/Ham’s F12 medium supplemented with 2% fetal bovine serum (FBS), l-glutamine (2 mM), HEPES (10 mM), insulin (5 µg/mL), transferrin (10 µg/mL), hydrocortisone (10 nM), and β-estradiol (10 nM) at 37 °C and 5% CO_2_.

### 2.3. Generation of Mouse Tracheal Epithelial Cell (mTEC) Cultures

Primary mouse tracheal epithelial cells (mTEC) cultures [36] were generated as previously described [37,38]. Briefly, tracheae were isolated from WT and *Tlr2^−/−^* mice under sterile conditions and processed to yield isolated mTECs as described. Isolated tracheal epithelial cells were cultured in the apical chamber of collagen-coated (rat tail collagen type I, 50 µg/mL, BD Biosciences) semi-permeable polycarbonate 0.4-µm pore membrane supports (Costar) in 6-, 12-, or 24-well plates with an appropriate volume of mTEC/Plus medium prepared as follows: mTEC/Basic medium (Dulbecco Modified Eagle Medium (DMEM)/Ham’s F-12 supplemented with 10% FBS, 15 mM HEPES, 4 mM glutamine, 3.6 mM NaHCO_3_, penicillin/streptomycin, and 250 ng/mL amphotericin B) with insulin (1 µg/mL; Sigma-Aldrich), transferrin (5 µg/mL; Sigma-Aldrich), cholera toxin (100 ng/mL; Sigma-Aldrich), epidermal growth factor (25 ng/mL; BD Biosciences), and bovine pituitary extract (30 µg/mL; PelFreeze). mTEC/Plus medium with added retinoic acid (RA; 50 nM) was added to the basal chamber before the addition of the cells to the apical chamber. Transmembrane resistance was measured with a volt/ohm meter (EVOM, World Precision Instruments) to assess confluence; the protocol used to generate an air–liquid interface (ALI) was initiated when transmembrane resistance TM > 1000 Ω cm^2^ was achieved. To establish an ALI, both apical and basal media were removed and mTEC/NuSerum (mTEC/Basic medium supplemented with 2% NuSerum (BD Biosciences) with 0.5 mM RA) was added to the basal chamber, leaving the apical chamber dry. Cells in the apical layer were washed with warm 1× phosphate-buffered saline (PBS) and the basal medium was changed every 2 days. Experiments in this study utilized mTECs cultured at day 5 after initiation of the ALI protocol unless otherwise stated.

### 2.4. Preparation of Virus Stocks and Titration by qPCR

Influenza A/FM/1/47-MA [39] was obtained from Dr. S. L. Epstein Food and Drug Administration/Center for Biologics Evaluation and Research (FDA/CBER) with permission from Dr. E. G. Brown (University of Ottawa, Canada). Influenza A/HK/1/68 used in experiments performed in vivo was obtained from Dr. J. D. Keicher (Symmune Therapeutics, Raleigh, NC, USA). Mouse-passaged stocks were prepared from both viruses. Tissue culture infectious dose (TCID_50_) and quantitative PCR titration were performed as previously described [38,40].

### 2.5. Preparation of Lactobacillus plantarum (Lp)

*Lactobacillus plantarum* NCIMB 8826 (Lp, ATCC BAA-793) was grown to stationary phase, heat-inactivated 70 °C for 30 min, washed once in sterile, endotoxin-free PBS, resuspended in PBS with 0.1% bovine serum albumin (BSA) at a concentration of 10^11^ cell equivalents/mL as previously described [21], and stored at −20 °C. Dilutions (10^7^, 10^8^, 10^9^, and 10^10^ cells/mL) were prepared in sterile, endotoxin-free PBS immediately before use.

### 2.6. Evaluation of Lp-Mediated Protection against Inf A Infection In Vivo

The first set of mice were anesthetized briefly with isoflurane and were inoculated with Lp (10^8^ cells in 50 µL) or PBS with 0.1% BSA diluent control on days −3 and −2, prior to virus challenge with 30 TCID_50_ units/5 µL on day 0. Body weights and survival were monitored daily. The second set of mice were inoculated as above with Lp or PBS diluent control, followed by virus or diluent alone on day 0. On day 8 post-virus challenge, mice were sacrificed and bronchoalveolar lavage (BAL) was performed (0.8 mL per lavage × 2 lavages = 1.4–1.6 mL per mouse). Virus titer (TCID_50_ units/50 µL BAL fluid) was determined as previously described [40]. Relative expression of proinflammatory mediators was evaluated by proteome profiling (Proteome Profiler Mouse Cytokine Array Kit, Panel A, R&D Systems, Minneapolis, MN, USA). Raw profiling data were normalized to expression levels observed in response diluent only (no Lp, no virus).

### 2.7. Responses of MLE-12 Cells to Administration of Lp and Pattern Recognition Receptor (PRR) Agonists

Low-passage MLE-12 cells were grown in DMEM/Ham’s F12 medium supplemented as described above in 24-well plates. Cultures at 75% confluence were washed with PBS and treated with Lp (10^6^ and 10^7^ cells/mL or diluent alone in 0.5 mL of growth medium). After 2 h at 32 °C and 5% CO_2_, Lp was removed; cells were then washed and provided with fresh growth medium. Supernatants were collected on the days indicated for analysis of proinflammatory cytokines by ELISA (DuoSet, R&D Systems). In similar experiments, MLE-12 cells were treated for 2 h with pattern recognition receptor (PRR) agonists (final concentration, 10 ng/mL; Invivogen), including Pam2CSK4 (TLR2/6), Pam3CSK4 (TLR1/2), MDP (NOD2), and L18-MDP (NOD2), or CL-429 (Pam2-MDP, dual TLR2/NOD2) or diluent control.

### 2.8. Treatment of mTECs with Lp and PRR Ligands

On day 5 after initiation of the ALI, the apical surfaces of mTEC cultures were washed with pre-warmed sterile PBS, followed by the addition of Lp (10^7^–10^10^/mL in 150 µL of PBS). After 2 h at 37 °C, in 5% CO_2_, Lp was removed from the apical surface and cells were washed with warm PBS. Medium harvested from the basal chamber every 24 h post-Lp treatment was evaluated by ELISA. In similar experiments, mTECs were treated with PRR agonists as described above (1, 10, and 100 ng/mL final concentrations) or with diluent control.

### 2.9. Infection of MLE-12 Cells with Inf A

To inoculate sub-confluent (75%) low-passage MLE-12 cells, 500 µL of a 1:50 dilution of live or heat-inactivated (95 °C for 5 min) stock suspension of Influenza A/FM/1/47-MA was diluted in of growth medium (at 10^5^ copies/µL, MOI = 0.5) was used. After 4 h at 32 °C at 5% CO_2_ with periodic rocking, the virus was removed, and cells were washed, provided with fresh growth medium, and returned to culture conditions as above. Cells were collected at 48 h, as indicated for the preparation of RNA to determine virus copy number as described below. Supernatants were collected every 24 h post-virus inoculation for evaluation of proinflammatory cytokines by ELISA.

### 2.10. Infection of mTECs with Inf A

To the apical surface of the mTEC culture, 150 µL of a 1:1000 dilution of a live or heat-inactivated stock suspension of Influenza A/FM/1/47-MA was added. Plates were incubated for 4 h at 32 °C under 5% CO_2._ The apical fluid was aspirated, and the cells were washed with warmed PBS. Then, 150 µL of fresh growth medium was added to the basal surface and the plates were returned to culture conditions as above.

### 2.11. Quantitative Evaluation of Virus Recovery by qRT-PCR

Total RNA was isolated from MLE-12 cells and mTECs using standard methods. Briefly, RNA-Bee (Tel-Test, Inc.) was added directly to the attached cells (MLE-12) or the apical chamber of the inserts (mTECs), followed by a 5-min incubation at RT. Cell extracts were equilibrated with chloroform (0.2× volume) and subjected to centrifugation in a microfuge. The resulting aqueous phase was collected, and RNA was purified using the RNeasy MiniElute Cleanup Kit (Qiagen). Purified RNA was eluted in minimal volumes of diethylpyrocarbonate (DEPC)-treated water (Quality Biologicals) and concentrations were determined using a NanoDrop One (Thermo Scientific). Approximately 1 µg of isolated RNA was treated with DNase I (Invitrogen). Samples were then reverse transcribed using the First Strand cDNA Synthesis Kit for RT-PCR (Roche) using random primers and no reverse transcriptase controls. Quantitative PCR reactions were carried out using a 7500 Real-Time PCR System (Applied Biosystems) under standard thermal cycling parameters with 25 µL of final volume including the reverse-transcribed cDNA, ABI TaqMan 2× Reagent (Thermo), and primer/probes specific for the Influenza A/FM/1/47-MA matrix protein (M1), including Forward Primer 5′-GCGAGGACTGCAGCGTAGAC-3′, Reverse Primer 5′-GGATCCCCGTTCCCATTAAG-3′, and Probe 6FAM-CTTTGTCCAAAATGCMGBNFQ, and primer/probes specific for Influenza A/HK/1/68, as previously described [38]. A VIC-labeled probe and primers that target mouse GAPDH were used for the normalization of all samples (ABI #43058313). Standard curves for M1 and GAPDH were generated for absolute quantification based on the amplification of a known quantity of 594 bp PCR amplicon of the M1 protein (GenBank ID KY348534.1) in the PCR2.1 vector and a mouse GAPDH plasmid pCMVpSport6 (ATCC #10539385), respectively. Results are reported as copies of M1 per copies of GAPDH × 10^5^.

### 2.12. Lp Priming Prior to Infection with Inf A

Lp priming of MLE-12 cells was performed on days −3 and −2 with 500-µL suspensions of *Lp* (10^7^ cells/mL), as described above. Following each 2-h incubation (32 °C and 5% CO_2_), cells were washed with PBS and re-fed with growth media. Lp- and diluent-primed cultures were inoculated with Inf A as described above. Lp priming of mTECs was performed via the addition of 150-µL suspensions containing 10^7^, 10^8^, 10^9^, or 10^10^ cells/mL to the apical surface. Following incubation for 2 h at 37 °C, 5% CO_2_, the Lp suspensions were aspirated carefully and the cells were washed with warm PBS before returning to culture conditions as described above. Lp- and diluent-primed cultures described above were followed by virus infection on day 0 as described. The cells were incubated at 32 °C, 5% CO_2_, with periodic rocking to maximize virus adsorption. After a 4-h incubation period, the virus suspension was carefully aspirated and the cells were washed with warm PBS. The medium in the basal chamber was replaced with fresh mTEC/NuSerum supplemented with retinoic acid at day 0 and every 48 h thereafter. RNA was harvested on day 0 (immediately after virus absorption), day 2, and day 4 after virus inoculation to assess replication by RT-qPCR. Medium from the basal chamber was harvested at 48 h (cumulative from d0 to d2 after virus inoculation) to evaluate concentrations of proinflammatory cytokines by ELISA.

### 2.13. Determination of Cytokine Concentrations in MLE-12 Supernatants and mTEC Basal Medium by ELISA

Supernatants from MLE-12 cells or medium collected from the basal chamber of mTEC cultures were aliquoted and stored at −20 °C until evaluation by ELISA. Quantitative analysis of critical cytokine mediators (CXCL1, CXCL10, CCL2, and CCL5) was performed by ELISA following the manufacturer’s instructions.

### 2.14. Flow Cytometry

CellStripper Non-Enzymatic Dissociation Reagent (Corning) was used to detach adherent MLE-12 cells from plates and mTECs from their membranes. This reagent was added to both the apical and basal chambers of mTEC cultures followed by an incubation period of 30 min at 37 °C and 5% CO_2_. The surfaces of the membranes supporting the mTEC cultures were gently washed with PBS supplemented with 10% FBS to recover any remaining cells. This process was repeated a second time to maximize cell recovery. After washing with PBS, the detached cells were stained with Live-Dead Aqua for 30 min at 4 °C followed by PBS with 0.1% BSA. Anti-CD16/CD32 (Mouse BD Fc Block; BD Biosciences) was added to each sample and control, followed by antigen-specific antibodies that target extracellular antigens. These antibodies (Abs) included APC anti-mouse CD326 (clone G8.8; Invitrogen), PE/Cy7 anti-mouse TLR4 (clone SA15-21; BioLegend), and PE anti-mouse TLR2 (clones CB225, 6C2, 11G5, mT2.7, and QA16A01, from BioLegend, Invitrogen, Novus, Invitrogen, and BioLegend, respectively). After a 30-min incubation with Abs at 4 °C, cells were washed with PBS with 0.1% BSA. For intracellular staining, cells were fixed and permeabilized using the eBioscience Intracellular Fix and Perm Set (Invitrogen). Antibodies used to detect intracellular antigens include AF700 anti-mouse NOD2 and FITC anti-rabbit NOD1 (both from Novus). Fluorescence minus one (FMO) control trials were performed to set gates. Experiments were performed using a BD LSRII flow cytometer and data were analyzed with FlowJo software.

### 2.15. Statistical Evaluation

Findings were evaluated using algorithms in GraphPad Prism 8.3 software. All experiments were repeated at least two times. Statistical significance was determined by Mann–Whitney U-tests or 1- or 2-way ANOVA, as appropriate. *p* values < 0.05 were interpreted as statistically significant.

## 3. Results and Discussion

### 3.1. Lactobacillus plantarum (Lp) Administered Directly to the Respiratory Tract Protects Mice against Inflammation and Weight Loss in Response to Infection with Inf A

**Figure 1:**
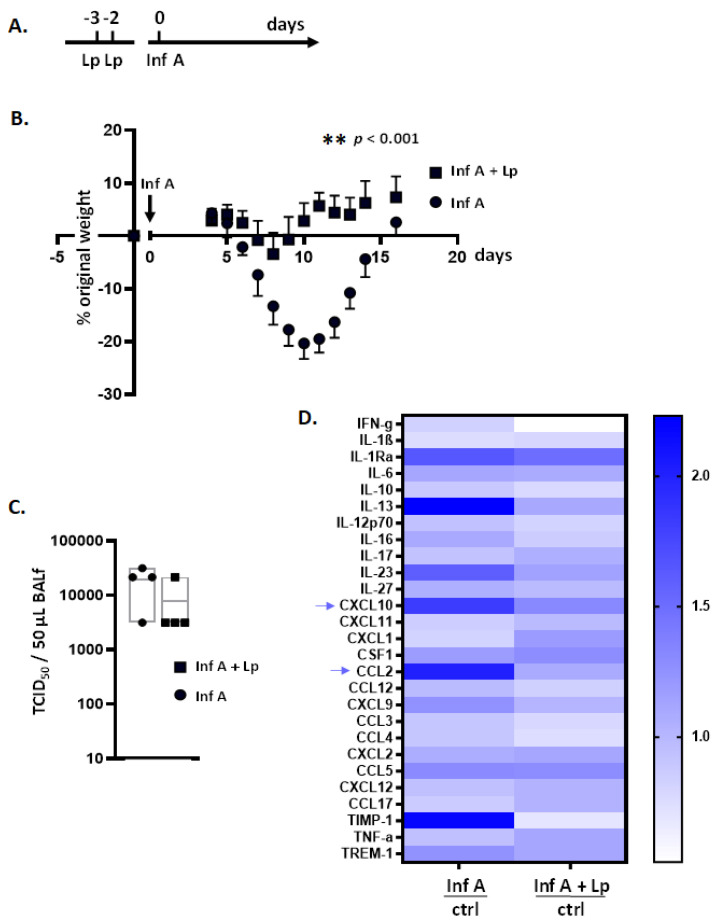
*Lactobacillus plantarum* (Lp) administered to the respiratory tract protects mice against inflammation and weight loss in response to infection with Influence (Inf) A. (**A**) Experimental protocol. Mice were inoculated intranasally with Lp (10^8^ cells in 50 µL) or diluent control on days −3 and −2 followed by Inf A (30 TCID_50_ units in 5 µL, 2.5 µL per nare) on day 0. (**B**) Weights on days 4–16 normalized to weights on day 0; *n* = 5 mice per group, ** *p* < 0.01 on days 7–14, two-way ANOVA. (**C**) Virus detected in BAL fluid on day 8 after priming with Lp or diluent alone followed by inoculation with Inf A, as per the protocol shown in (**A**). (**D**) Heat map documenting the results of cytokine profiling of pooled BAL fluid collected from mice primed with Lp or diluent alone followed by inoculation with Inf A or diluent alone (*n* = 5 mice in each of four groups), as per the protocol shown in (**A**) and evaluated on day 8. Raw data for each cytokine mediator were normalized to the diluent/diluent (i.e., no Lp and no Inf A); *p* < 0.05.

As neither MLE-12 cells nor mTECs were susceptible to infection with PVM, we focused this study on inflammatory responses to infection with Inf A. For this aim, we primed mice with Lp or a diluent control followed by inoculation with a sublethal dose of Inf A (Figure 1A) and measured weight loss and local inflammatory responses. Inf A infection alone resulted in a maximum weight loss of 20 ± 3% at day 10 post-inoculation. By contrast, mice primed with Lp prior to inoculation with Inf A experienced minimal weight loss, reaching a maximum of 3.4 ± 4% on day 8 (*p* < 0.001 (Figure 1B)). Consistent with our results with the PVM infection model, Lp priming had no impact on virus titer (Figure 1C) but did suppress virus-induced inflammation. As shown in Figure 1D, numerous proinflammatory mediators were produced in response to Inf A infection alone; Lp priming on days −3 and −2 suppressed this response (*p* < 0.05). These results largely parallel those reported previously for mice primed with *Lactobacillus* spp. followed by infection with Inf A [27,29], RSV [28], or PVM [21,22,23,24,25,26].

### 3.2. MLE-12 Cells and mTECs Support Replication of Inf A and Produce Proinflammatory Cytokines in Response to Virus Infection

**Figure 2:**
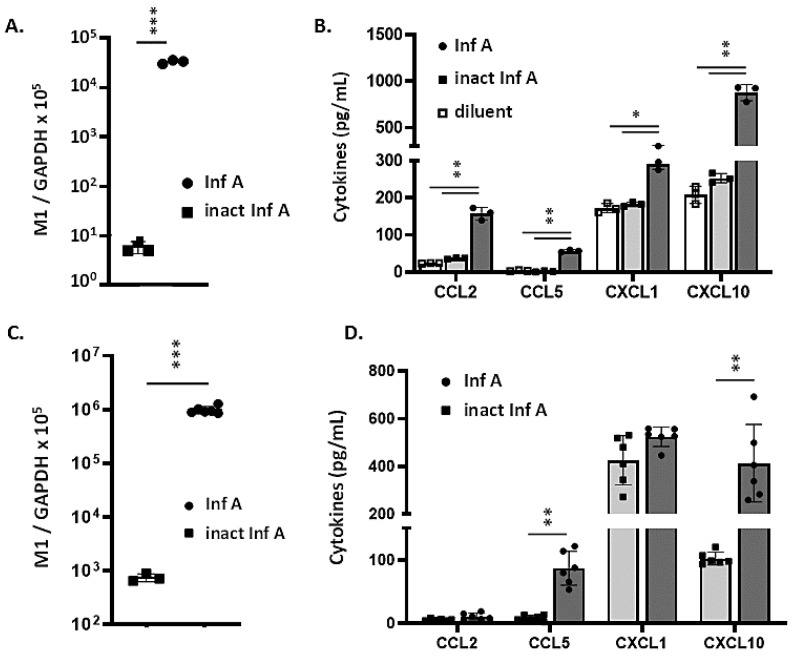
MLE-12 cells and mouse tracheal epithelial cells (mTECs) support replication of Inf A and produce proinflammatory cytokines in response to infection. (**A**) Virus detected in MLE-12 cells at 48 h after inoculation with live or heat-inactivated Inf A (4 h, 32 °C, MOI = 0.5); *** *p* < 0.001, Mann–Whitney U-test. (**B**) MLE-12 cells infected with live Inf A produce and release proinflammatory cytokines, including CCL2, CCL5, CXCL10, and CXCL1, as shown; * *p* < 0.05, ** *p* < 0.01, one-way ANOVA. (**C**) Virus detected in mTECs at 48 h after inoculation with live or heat-inactivated Inf A; *** *p* < 0.001, Mann–Whitney U-test. (**D**) mTECs infected with live Inf A produce and release proinflammatory cytokines, including CCL5 and CXCL10, but not CCL2 or CXCL1, as shown; ** *p* < 0.01, one-way ANOVA.

To examine the direct impact of Lp on respiratory epithelial cells, we generated cell culture models using MLE-12 cells and mTECs. MLE-12 cells are derived from a primary mouse lung adenocarcinoma and display characteristics of type II alveolar epithelial cells [35]. MLE-12 cells were challenged with live or non-replicating heat-inactivated Inf A Multiplicity of infection (MOI = 0.5); virus recovery was evaluated by qRT-PCR targeting the virus M1 gene at 24 h after inoculation. While we detected few to no virion copies (6.0 ± 1.6 [copies M1/copies GAPDH] × 10^5^) in cells from cultures that were inoculated with heat-inactivated virus, we detected 34,000 ± 3000 copies ([M1/GAPDH] × 10^5^) in cells inoculated with live virus (~5600-fold difference, *p* < 0.001; (Figure 2A)). This finding is consistent with previous reports documenting infection of MLE-12 cells with Inf A [41,42,43]. Likewise, challenge with live virus, but not with heat-inactivated virus, resulted in the production and release of proinflammatory cytokines, notably CCL2, CCL5, CXCL1, and CXCL10 (Figure 2B). We have focused on outcomes associated with these specific cytokines as they have been featured in reports documenting proinflammatory responses to Inf A, RSV, PVM, and SARS-CoV-2 [8,9,10,11,12,13,14,15,44].

Polarized mouse tracheal epithelial cell (mTEC) cultures also supported replication of Inf A (Figure 2C). However, infection of mTECs with replicating Inf A resulted in the production and release of CCL5 and CXCL10 only; little to no CCL2 or CXCL1 was detected over levels observed in response to heat-inactivated virus (Figure 2D).

In a previous study, we presented immunohistochemical data documenting infection of bronchial epithelial cells at day 6 after inoculation with Influenza A [38]. The findings presented here are also consistent with those published previously by Weinheimer et al. [45] who presented immunohistochemical data documenting Influenza A infection in human type II pneumocytes and those of Ibricevic et al. [46] who used a similar strategy to document Influenza A infection of type II alveolar epithelial cells in vivo; the latter publication also documented infection of mTECs via this method.

Likewise, while the three aforementioned chemokines all contribute to the antiviral inflammatory response, their individual signaling pathways and roles in promoting cellular recruitment may differ from one another. Most notably, each chemokine interacts with a distinct CC or CXC chemokine receptor expressed on one or more cells of both myeloid and lymphoid lineages. Although specific outcomes have been defined in controlled experiments carried out ex vivo, cross-activation and modulation of these pathways in the setting of an influenza-associated cytokine storm [47,48,49,50] may ultimately blur the lines defining distinct activation and response pathways. The inter-dependent role(s) of each cytokine might ultimately emerge from experiments that feature Inf A infection with single receptor blockade and/or in ligand- or receptor gene-deleted mice [51,52,53,54].

### 3.3. Detection of Critical Pattern Recognition Receptors (PRRs) in MLE-12 Cells and mTECs by Flow Cytometry

After characterizing the biochemical inflammatory response to Inf A infection, we then determined whether one or both of these cell culture models was capable of effective PRR-mediated interactions with Lp. In previous work, we found that Lp activated intracellular signaling via interactions with TLR2 and NOD2 [24]. This finding was not unexpected, given that Lp is a Gram-positive bacterial species with cell wall-associated lipoteichoic acids and muramyl dipeptides that have been characterized as TLR2 and NOD2 ligands, respectively [55,56,57]. We also found that administration of the bifunctional TLR2-NOD2 ligand, CL-429, protected mice from the lethal sequelae of PVM infection and reduced virus-induced inflammatory responses when administered to the airways of wild-type mice; similarly, NOD2-TLR2 gene-deleted mice did not respond effectively to Lp priming [24].

**Figure 3:**
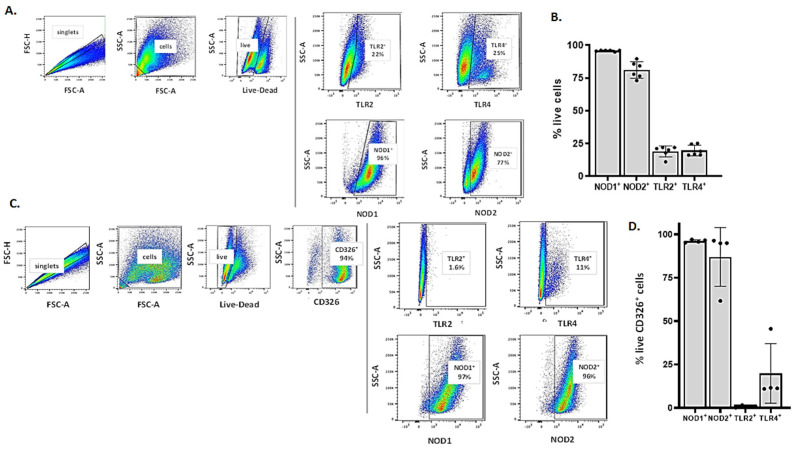
Detection of critical pattern recognition receptors (PRRs) in MLE-12 cells and mTECs by flow cytometry. (**A**) Strategy used to detect TLR2, TLR4, NOD1, and NOD2 expression in MLE-12 cells. (**B**) Quantitative analysis of PRR expression as % live cells. (**C**) Strategy used to detect the expression of TLR2, TLR4, NOD1, and NOD2 in CD326^+^ mTECs. (**D**) Quantitative analysis of PRR expression as % live CD326^+^ cells.

The flow cytometry strategy used to detect cell surface and intracellular pattern recognition receptors in MLE-12 cells is shown in Figure 3A. Nearly all (>95%) of the MLE-12 cells were NOD1^+^ and 81% ± 6.3% expressed NOD2. By contrast, 19% ± 4% and 20% ± 4% expressed TLR2 and TLR4, respectively (Figure 3B). The flow cytometry strategy used to detect these PRRs in mTECs is shown in Figure 3C; of note, nearly all mTECs (94%), but virtually none of the MLE-12 cells (data not shown), express CD326, also known as EpCAM, which is an epithelial cell-specific transmembrane glycoprotein and homotypic cell adhesion molecule [58]. Similar to our findings for MLE-12 cells, nearly all of the mTECs (>96%) were NOD1^+^; likewise, 87% ± 17% were NOD2^+^ and 20% ± 17% were TLR4^+^. By contrast, very few of the mTECs expressed TLR2 (0.93 ± 0.58%; (Figure 3D)). This finding was confirmed with additional anti-TLR2 clones and mTECs derived from *Tlr2^−/−^* mice (Supplemental Figure S1).

### 3.4. MLE-12 Cells Respond to Lp and TLR2 Agonists

**Figure 4:**
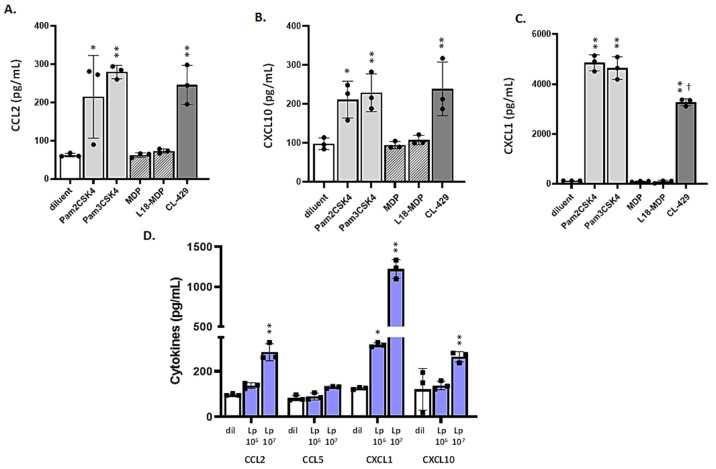
MLE-12 cells respond to Lp and TLR2 agonists. MLE-12 cells release pro-inflammatory mediators, including (**A**) CCL2, (**B**) CXCL10, and (**C**) CXCL1 at 24 h after a 2-h treatment with TLR2 ligands, Pam2CSK4 and Pam3CSK4, and the combined TLR2-NOD2 ligand CL-429 (Pam2C-MDP). No cytokine production was observed in response to the administration of the NOD2 ligands MDP or L18-MDP. All biochemical ligands were added to cultures at final concentrations of 10 ng/mL. (**D**) MLE-12 cells release pro-inflammatory mediators, including CCL2, CXCL1, and CXCL10, but not CCL5, at 24 h after a 2-h treatment with increasing concentrations of Lp (10^6^ or 10^7^/mL); * *p* < 0.05 or ** *p* < 0.01 *vs.* diluent alone; ^†^ *p* < 0.05 vs. Pam2CSK4, one-way ANOVA.

To assess the functionality of these two PRRs, we examined MLE-12 cell responses to characterized TLR2 and NOD2 agonists. We found that MLE-12 cells produce and release proinflammatory cytokines, including CCL2, CXCL10, and CXCL1, in response to administration of the diacylated lipopeptide TLR2 agonists Pam2CSK4 and Pam3CSK4. Interestingly, no responses were observed to the administration of the NOD2 ligands MDP or L18-MDP, despite the strong expression of this PRR in MLE-12 cells. MLE-12 cells also responded to the bi-functional agonist CL-429 (Pam2-MDP); as might be anticipated, the responses to CL-429 largely replicated those of the TLR2 ligands (Figure 4A–C). MLE-12 cells also produce and release CCL2, CXCL1, and CXCL10 (but not CCL5) in a dose-dependent fashion in response to Lp (Figure 4D). These results are analogous to those reported by Garcia-Crespo et al. [33], who found that administration of Lp to the respiratory tract resulted in the rapid and transient production of proinflammatory chemokines in vivo as part of its protective mechanism. While there are several previous publications documenting responses of MLE-12 cells to TLR2 agonists [59,60], to the best of our knowledge, this is the first dataset in which responses to MDP, L18-MDP, and the bifunctional CL-429 ligand were examined.

### 3.5. Differential Responses of mTECs to TLR2 Ligands and CL-429

**Figure 5:**
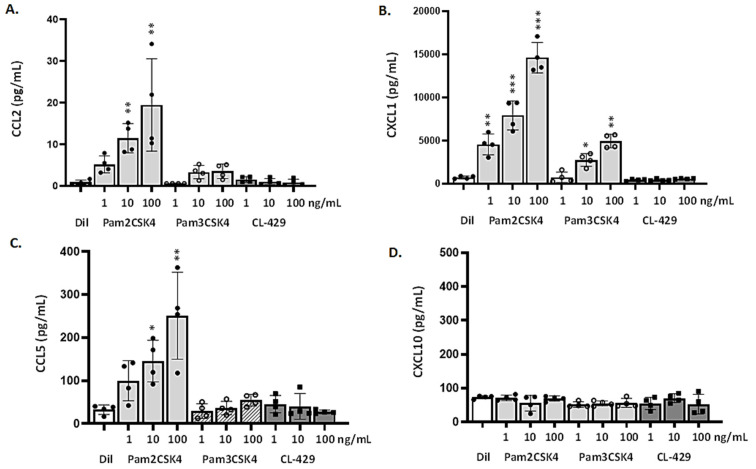
mTECs exhibit distinct responses to the TLR2 agonists Pam2CSK4, Pam3CSK4, and the combined TLR2-MDP ligand, CL-429. mTECs release proinflammatory mediators, including (**A**) CCL2, (**B**) CXCL1, and (**C**) CCL5, but not (**D**) CXCL10, at 48 h after treatment for 2 h with the TLR2/1 ligand Pam2CSK4. Minimal responses were observed in response to administration of Pam3CSK4 (production and release of CXCL1 only). No responses were observed to CL-429 or in response to the NOD2 ligands MDP and L18-MDP (data not shown). All biochemical ligands were added to cultures at final concentrations of 1, 10, or 100 ng/mL. * *p* < 0.05, ** *p* < 0.01, or *** *p* < 0.001 *vs.* diluent alone, one-way ANOVA.

Although the TLR2^+^ population is comparatively small, mTECs are, nonetheless, capable of responding to TLR2 agonists, albeit somewhat differently than the MLE-12 cells. mTECs respond primarily to Pam2CSK4 (a ligand for the TLR2/6 heterodimer, discussed further below) which elicits the dose-dependent production and release of CCL2, CXCL1, and (in contrast to MLE-12 cells), CCL5 (Figure 5A–C). By contrast, responses to Pam3CSK4 (a ligand for the TLR2/1 heterodimer) were comparatively limited. Specifically, administration of Pam3CSK4 to mTECs in concentrations of 10 and 100 µg/mL elicited production and release of limited quantities of CXCL1 and little to no CCL2; no statistically significant release of CCL5 was detected in response to any of these ligands (Figure 5D). Similar to MLE-12 cells, mTECs do not produce or release any of these pro-inflammatory cytokines in response to NOD2 ligands (data not shown). In contrast to results from MLE-12 cells, no cytokine production or release was observed in response to the bifunctional ligand CL-429. Of note, no CXCL10 was detected in mTEC cultures in response to any ligand, even at the highest concentrations used.

We note with interest the fact that mTECs are capable of responding to the diacylated lipopeptide ligand Pam2CSK4 despite minimal expression of TLR2 (Figure 3 and Supplementary Material Figure S1). While TLR6, the receptor that forms active heterodimers with TLR2, is clearly expressed in respiratory epithelial cells (reviewed in [61]), there is no evidence in the literature suggesting that diacylated lipopeptide ligands have any capacity to bypass TLR2. Specifically, Takeuchi et al. [62] found that while TLR6^−/−^ cells were unable to respond to the diacylated lipopeptide ligand macrophage-activating lipopeptide-2 (MALP-2), reconstitution experiments performed in double-deleted (TLR2^−/−^TLR6^−/−^) cells revealed that ligand-mediated signaling required concomitant expression of both receptors.

We were also intrigued to find that neither MLE-12 cells nor mTECs were capable of generating responses to NOD2 agonists. This is an intriguing point that merits further consideration. First, we know that the NOD2 agonist MDP can be fully effective when used to activate target cells ex vivo. Results from our previous studies revealed that both the MH-S alveolar macrophage cell line [63] and alveolar macrophages (AMs) from wild-type but not NOD2/TLR2 gene-deleted mice [24] are fully capable of producing proinflammatory cytokines in response to MDP administered at 10 µg/mL, as shown here. One critical point that might be recognized is that evaluation by flow cytometry provides insight primarily into the number of cells detected using a given antibody (i.e., percent positive). This does not necessarily reflect the number of immunoreactive receptors per cell or their capacity to respond to a given ligand. As such, although more than 90% of the cells were identified NOD2^+^, these receptors may exist in a conformation or intracellular localization rendering them incapable of responding positively to this ligand. Of note, Maekawa et al. [64] documented an inactive form of NOD2 based on their elucidation of its crystal structure. Likewise, as noted above, although mTECs produced proinflammatory cytokines in response to Pam2CSK4, they did not respond at all to CL-429, a bifunctional ligand that includes the diacylated Pam2 moiety ligated directly to the NOD2 agonist, MDP. Taken together, these results suggest that the NOD2 ligands may counteract TLR2-mediated responses specifically in mTECs. These results are similar to those described previously in mouse models of colitis [65] and human monocytes [66].

### 3.6. mTECs from Wild-Type (WT) but Not Tlr2^−/−^ Mice Respond to Lp

**Figure 6:**
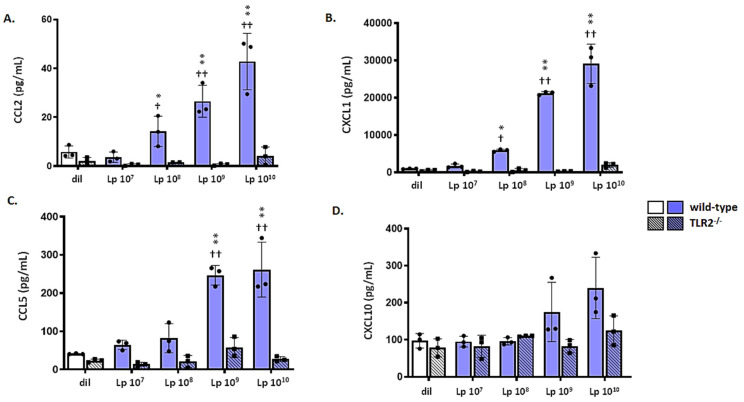
mTECs from wild-type (WT) but not *Tlr2*^−/−^ mice respond to Lp. mTECs from WT mice release proinflammatory mediators, including (**A**) CCL2, (**B**) CXCL1, (**C**) and CCL5, but not (**D**) CXCL10, at 48 h after treatment for 2 h with Lp (10^7^–10^10^ cells/mL). No responses to Lp were observed from mTECs derived from *Tlr2*^−/−^ mice, * *p* < 0.05 and ** *p* < 0.01 vs. responses from cells from *Tlr2^−/−^* mice; ^†^ *p* < 0.05 and ^††^ *p* < 0.01 vs. responses of cells from wild-type mice to lower concentrations of Lp.

mTECs from wild-type mice also respond to Lp and largely follow the same pattern observed for Pam2CSK4. Administration of Lp results in the dose-dependent production and release of CCL2, CXCL1, and CCL5 in mTECS from wild-type but not *Tlr2*^−/−^ mice (Figure 6A–C). Analogous to the results shown in Figure 5, we observed no production or release of CXCL10 (Figure 6D).

### 3.7. Lp Does Not Elicit Cross-Protection against Virus-Induced Inflammation

**Figure 7:**
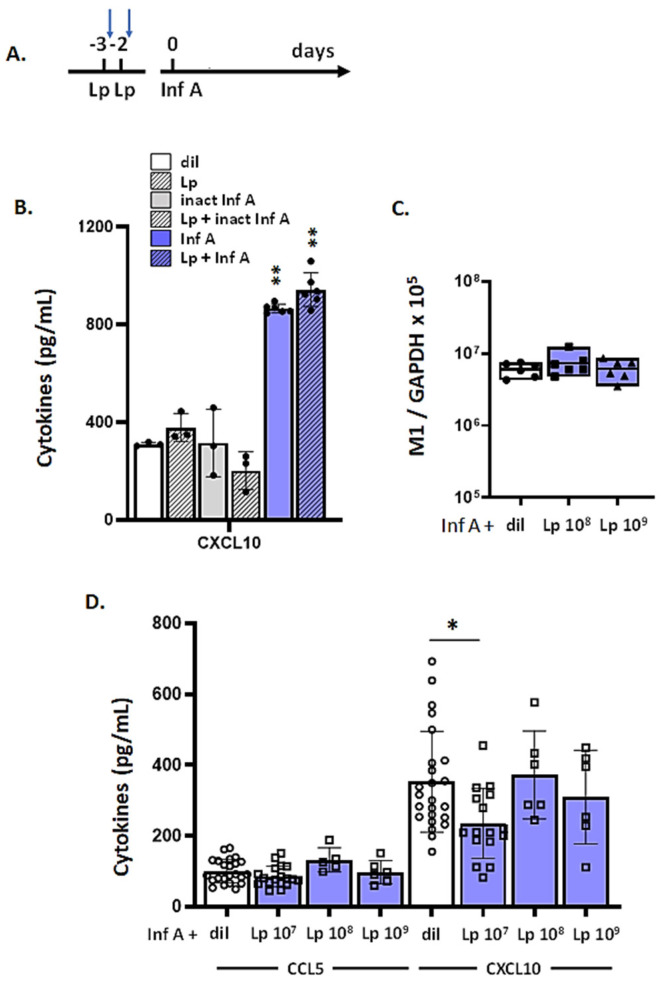
Treatment with Lp does not elicit cross-protection against virus-induced inflammation in either MLE-12 or mTEC cultures. (**A**) Experimental protocol. Cell cultures were treated with Lp or diluent control on days indicated followed by Inf A on day 0; blue arrows indicate extensive washing at 2 h post-treatment. (**B**) Proinflammatory cytokines CCL5 and CXCL10 detected in culture supernatants from MLE-12 cells at 48 h after inoculation with live or heat-inactivated Inf A as in (**A**), ** *p* < 0.01 vs. diluent alone, one-way ANOVA. (**C**) Virus detected in mTECs pretreated with diluent or Lp (10^8^ or 10^9^ cells/mL) at 48 h after inoculation with live Inf A on day 0. (**D**) Proinflammatory cytokines detected in supernatants of diluent or Lp-primed mTECs at 48 h after inoculation with live Inf A; * *p* < 0.05 vs. diluent alone, one-way ANOVA.

We then treated both MLE-12 and mTECs using a protocol that was designed to replicate conditions that elicited Lp-mediated cytokine suppression in vivo (Figure 7A). Specifically, cell cultures were treated with Lp for 2 h each on days −3 and −2; each treatment was followed by extensive washing to replicate the effects of endogenous clearance [33]. On day 0, cultures were washed and inoculated with Inf A and evaluated at 48 h thereafter. Similar to findings obtained in vivo [33], no ongoing cytokine production or release over baseline levels was detected at this time point. Results from the administration of Lp to MLE-12 cells followed by Inf A infection are shown in Figure 7B. Consistent with the findings shown in Figure 2B and Figure 4B, Inf A infection alone resulted in the production and release of CXCL10. We found that priming with Lp on days −3 and −2 did not suppress this response. Similar results were obtained for CCL5 (data not shown).

Results of Lp priming followed by Inf A infection of mTECs are shown in Figure 7C,D. Lp priming had no impact on virus replication and little to no impact on the production and release of CCL5 and CXCL10.

### 3.8. Discussion

In this manuscript, we evaluated the interactions between Lp and Inf A infection in two mouse respiratory epithelial cell culture models. Although both MLE-12 cells and polarized mTECs express relevant PRRs, exhibit PRR-dependent inflammatory responses to Lp, and are readily infected with Inf A, neither culture system adequately replicated Lp-mediated suppression of virus-induced inflammation as observed in vivo. These findings are notable given that Neagos et al. [60] reported that MLE-12 cells become cross-tolerant and, thus, unable to mount inflammatory responses after repeated administration of PRR agonists, including Pam3CSK4. Taken together, these results suggest that the factors underlying Lp-mediated suppression of virus-induced inflammation are more complex and are likely to involve additional, potentially unrelated molecular mechanisms.

Many of our previous studies focused on Lp-mediated suppression of virus-induced inflammation in studies performed in vivo with the rodent pneumovirus pathogen PVM. We attempted to establish PVM infection in both MLE-12 cells and mTECs using a variety of strategies and detection methods, including our standard qRT-PCR assay [67] as well as immunofluorescence microscopy and flow cytometry, with the latter two methods featuring mKATE-tagged recombinant virus [68]. As described previously, PVM can infect mouse bronchial epithelial cells [69] and alveolar macrophages [68] in vivo. We have also established PVM infections in mouse macrophage (RAW 264.7) [70] and hamster fibroblast (BHK) cell lines [68]. As such, it is not at all clear why MLE-12 cells and mTECs are resistant to infection; however, similar findings resulted when we explored PVM infection in the mouse alveolar macrophage (MH-S) cell line [63].

Although epithelial cells can clearly respond to Lp and, likewise, are susceptible to virus infection, our findings suggest that Lp-mediated cytokine suppression may involve interactions with other cells, potentially those recruited to the airways. Although previous results from in vivo experiments have ruled out critical roles for B cells, T cells, and recruited monocytes (i.e., in *Ccl2*-deficient mice) [22,25,26], we have not fully examined the role of neutrophils and neutrophil mediators and their direct interactions with the respiratory epithelium. Lp priming promotes neutrophil recruitment to the airways [33], as does infection with Inf A [39,71]; interactions between respiratory epithelial cells and immune cells, including neutrophils, have been documented in the literature [72,73,74]. Although we were unable to carry out these experiments in vivo with neutrophil-deficient or neutrophil-depleted mice ([33]; Garcia-Crespo, K. E. and Rosenberg, H. F., unpublished data), this issue might be addressed with future experiments in cell culture models.

Lp-mediated suppression of virus-induced inflammation may involve responses from PRRs other than (or in addition to) TLR2 and NOD2. While administration of CL-429 (a bifunctional TLR2-NOD2 agonist) to the respiratory tract promotes protection against virus-induced inflammation, our findings with *Tlr2-Nod2* gene-deleted mice do not rule out critical contributions from other receptors. While TLR2- and NOD2-mediated responses to bacteria in the respiratory tract have been characterized extensively [75,76,77], we cannot rule out contributions from alternative signaling mechanisms such as those mediated by peptidoglycan recognition proteins (PGLYRPs; [78,79]). Of note, PGLYRP1 has been detected in both airway epithelial cells and neutrophil granules [80,81].

These experiments represent a direct extension of our studies that address the critical contributions of inflammation to the clinical outcome of respiratory virus infection. Our earlier studies revealed that the inflammatory response plays a critical role in determining the clinical outcome of infection and that virus-induced inflammation cannot be fully suppressed by administration of replication inhibitors [18,19,21,23,82]. Likewise, given the multifactorial nature of the cytokine storm, blockade of a single cytokine or cytokine receptor signaling pathway may not be fully effective at suppressing virus-induced inflammation; this has been made all too apparent in cases of severe and fatal infection with pandemic SARS-CoV-2 [83,84]. Creative and far-reaching approaches may be needed to combat virus-induced inflammation and to develop effective treatment strategies for use both now and during any future emerging pandemics.

## Supplementary Materials

The following are available online at https://www.mdpi.com/1999-4915/13/1/2/s1, Figure S1: Further efforts to detect TLR2 expression in mTECs.
