# Supplementary material for: Respiratory Epithelial Cells Respond to Lactobacillus plantarum but Provide No Cross-Protection against Virus-Induced Inflammation
Source: Viruses. 2020 Dec 22;13(1):2. doi: 10.3390/v13010002 (PMC7821944; doi:10.3390/v13010002)
Supplement: Supplementary file 1 [file viruses-13-00002-s001.pdf]

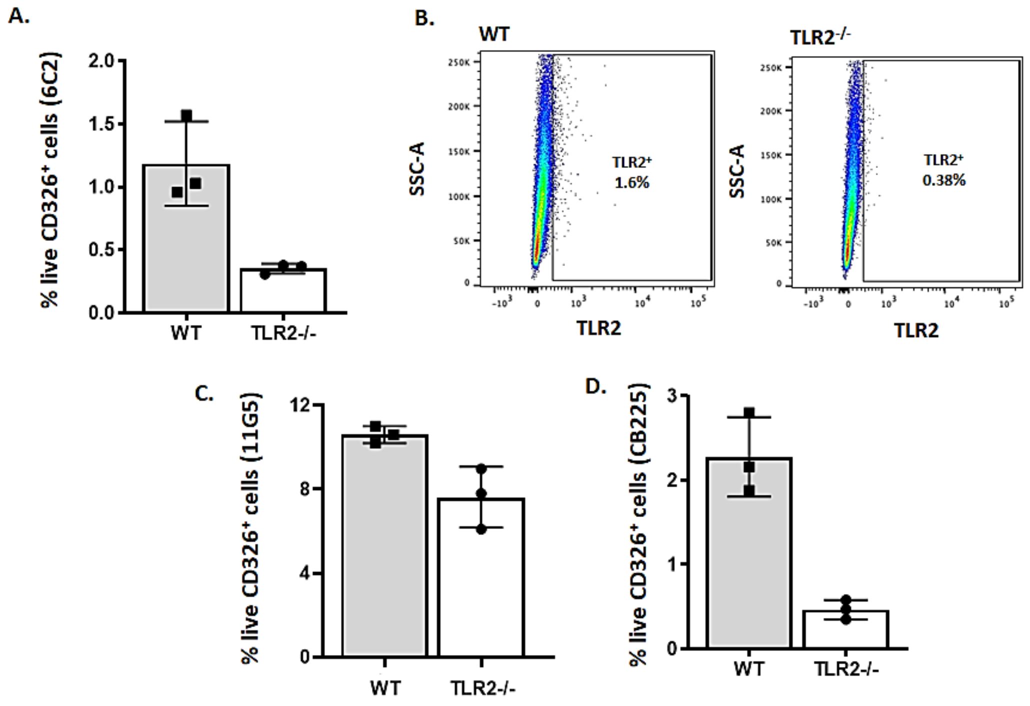

Suppl. Fig. 1. Further efforts to detect TLR2 expression in mTECs.

Expression of TLR2 was evaluated further by flow cytometry using multiple anti-TLR2 antibody clones and mTEC cultures prepared from both WT and *Tlr2*$^{-/-}$ mice. **(a)** Percentage live CD326^{+} cells detected with the anti-TLR2 clone, 6C2 (see also Figure 3). **(b)** Flow plots documenting sample results as shown in (a), **(c)** Percentage of live CD326^{+} cells detected with the anti-TLR2 clone, 11G5. **(d)** Percentage of live CD326^{+} cells detected with the anti-TLR2 clone CB225.
